# Viroporins in the Influenza Virus

**DOI:** 10.3390/cells8070654

**Published:** 2019-06-29

**Authors:** Janet To, Jaume Torres

**Affiliations:** School of Biological Sciences, Nanyang Technological University, 60 Nanyang Drive, Singapore 637551, Singapore

**Keywords:** influenza virus, matrix protein 2 (M2), viroporins, ion channel inhibition, protein–protein interactions

## Abstract

Influenza is a highly contagious virus that causes seasonal epidemics and unpredictable pandemics. Four influenza virus types have been identified to date: A, B, C and D, with only A–C known to infect humans. Influenza A and B viruses are responsible for seasonal influenza epidemics in humans and are responsible for up to a billion flu infections annually. The M2 protein is present in all influenza types and belongs to the class of viroporins, i.e., small proteins that form ion channels that increase membrane permeability in virus-infected cells. In influenza A and B, AM2 and BM2 are predominantly proton channels, although they also show some permeability to monovalent cations. By contrast, M2 proteins in influenza C and D, CM2 and DM2, appear to be especially selective for chloride ions, with possibly some permeability to protons. These differences point to different biological roles for M2 in types A and B versus C and D, which is also reflected in their sequences. AM2 is by far the best characterized viroporin, where mechanistic details and rationale of its acid activation, proton selectivity, unidirectionality, and relative low conductance are beginning to be understood. The present review summarizes the biochemical and structural aspects of influenza viroporins and discusses the most relevant aspects of function, inhibition, and interaction with the host.

## 1. Introduction

### 1.1. Influenza Viruses

Influenza viruses are enveloped, segmented, negative-sense RNA viruses belonging to the Orthomyxoviridae family. Four influenza virus types have been identified to date, classified based on their core proteins: A, B, C [1] and D [2,3]. Type A is known to infect humans, as well as porcine, bovine and canine species [4]. The main reservoir of the influenza A viruses are aquatic birds [5]. The seasonal flu caused by influenza A virus (IAV) in humans is a very contagious respiratory illness that is among the top ten leading causes of death in the United States and associated with high medical burden [6,7,8]. Sudden pandemics with high mortality rates are caused by the sporadic transmission of avian or swan influenza viruses to humans, as pre-immunity to these new strains is non-existent [6,7]. Influenza B virus (IBV), its close relative, is less severe but still capable of causing serious outbreaks, is responsible for seasonal influenza epidemics among humans [9,10], and accounts for half of the influenza diseases in recent years (www.cdc.gov). Types B (IBV) and C (ICV) infect humans and pigs [11], whereas type D (IDV) infects cattle and pigs [3]. In addition, IBV has also been reported to infect harbor seals [12]. Although IDV infection in cattle is usually asymptomatic, it can lead to disease in swine [2,13].

### 1.2. The Proteins in Influenza Viruses

In IAV, the viral envelope contains cholesterol-enriched lipid rafts and transmembrane glycoproteins hemagglutinin (HA) and neuraminidase (NA) [14], which are used to further classify IAV strains. A third membrane protein is matrix protein 2 (AM2 in IAV), present in lower abundance [15]. HA, NA, and M2 are type I, type II, and type III membrane proteins, respectively [16,17,18]. A layer of matrix protein 1 (M1) just underneath the membrane is the most abundant protein of the virus. M1 forms an internal coat, enclosing the viral ribonucleoproteins as well as three polymerase proteins (PA, PB1, and PB2) that form the viral RNA polymerase complex [19]. 

IAV and IBV have eight negative-sense RNA segments, each encoding one or two viral proteins. IAV encodes 10–11 proteins: HA, NA, NP, M1, M2, PA, PB1, PB2, NS1, NS2, and PB1-F2 [19]. IBV has the same proteins as IAV, except for the PB1-F2 protein, and has an additional surface glycoprotein NB that is unique to flu B. ICV and IDV consist of only seven gene segments each, and they do not encode envelope glycoproteins HA and NA. Instead, they carry only one glycoprotein, HEF (haemagglutinin–esterase fusion), which combines the functions of HA and NA [20]. 

M2, PB1-F2, and NB proteins are classified as viroporins, i.e., virus-encoded short polypeptides (approximately 60–120 amino acids) that have one, two, or even three [21] transmembrane (TM) α-helical domains. These polypeptides form oligomers of typically four to six monomers that permeabilize membranes to ions [22,23,24]. 

## 2. AM2 and BM2

### 2.1. AM2

The RNA segment 7 from IAV encodes both the M1 protein and AM2 proton channel via alternative splicing [25,26]. AM2 is 97-residues long and forms a channel assembled by the tetramerization of four left-handed TM helices stabilized by disulfide bridges [18,27]. The AM2 monomer consists of an N-terminal ectodomain (residues 1–24), an α-helical (TM) domain (residues 25–46), and a C-terminal cytoplasmic tail (CT) domain (residues 47–97) that contains a highly conserved amphipathic helix (APH) (residues 51–59) [25,28].

AM2 forms an acid-activated proton channel with an asymmetrical preference of proton conduction from the viral exterior (N-terminus) to the interior (C-terminus). The proton channel activity of AM2 is essential for two important roles. Upon viral entry into cells via sialic acid-containing receptor-mediated endocytosis, the low pH (5 to 6) of late endosomes activates the proton channel activity of AM2, which acidifies the viral interior [25] with subsequent detachment of M1 from the vRNP complex. This enables trafficking of vRNPs into the host cell nucleus for synthesis of mRNA and vRNA [29,30]. The channel activity of M2 is also crucial during viral maturation, where it equilibrates the pH across the trans-Golgi membrane to prevent the viral HA from undergoing premature conformational change while being transported to the plasma membrane of infected cells [31,32,33,34]. Conductance and inhibition studies have used the transmembrane domain of AM2 (AM2-TM), since in *Xenopus* oocytes AM2(21–51) has retained the proton selectivity and drug sensitivity observed in full-length AM2 protein [35]. 

**Figure 1:**
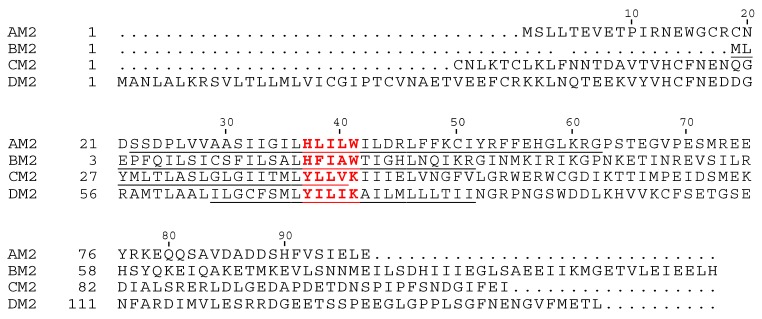
Sequence alignment of M2 viroporins in influenza. Influenza A M2 (AM2; strain A/Udorn/1972 H3N2), influenza B M2 (BM2; strain B/Taiwan/70061/2006), influenza C M2 (CM2; strain C/Ann Arbor/1/1950) and influenza D M2 (DM2; strain D/swine/Oklahoma/1334/2011). The predicted transmembrane regions are underlined. The functional motifs HxxxW (in AM2 and BM2) and YxxxK (in CM2 and DM2) are indicated in bold red font. Numbering corresponds to AM2. Sequences were retrieved from UniProt (www.uniprot.org).

Four main features characterize AM2-facilitated membrane permeabilization to protons: acid activation, proton selectivity, relative low proton conductance, and unidirectionality. These features are encoded in key pore-lining residues of the AM2 channel. AM2 has a conserved HxxxW functional motif in its TM domain (Figure 1), where His37 is responsible for proton selectivity and acid activation [36], and Trp41 ensures asymmetric proton conduction from the N-terminus to the C-terminus [37]. Other residues surrounding this motif also contribute to the dynamics and proton transfer equilibria in the channel. 

### 2.2. BM2

In IBV, RNA segment 7 encodes both M1 protein and BM2 [38,39]. Like AM2, BM2 is a pH-activated proton channel [40] and has a similar monomeric and oligomeric organization as described above for AM2 [41,42]. Like AM2, a truncated peptide containing its TM, BM2(1–33), conducts protons when incorporated into artificial liposomes [42] and *Xenopus* oocytes, with similar conductance and proton-selectivity as that observed in full-length BM2 protein [43]. Despite these similarities, AM2 and BM2 share almost no sequence identity, with the exception of an HxxxW motif in the TM domain (see Figure 1), which may explain some of their common features. AM2 and BM2 also differ in post-translational modifications; while BM2 is only modified by phosphorylation [44], AM2 contains disulfide bonds and is palmitoylated and phosphorylated [45,46,47,48]. Like AM2, BM2 is essential for virus uncoating in the endosome and for pH equilibration between Golgi lumen and cytoplasm during virus protein transport [38]. However, while the AM2 ectodomain is important for its incorporation into virions [49], the BM2 has only a small ectodomain [41,42] (Figure 1). 

### 2.3. Acid Activation Mechanism of AM2

**Figure 2:**
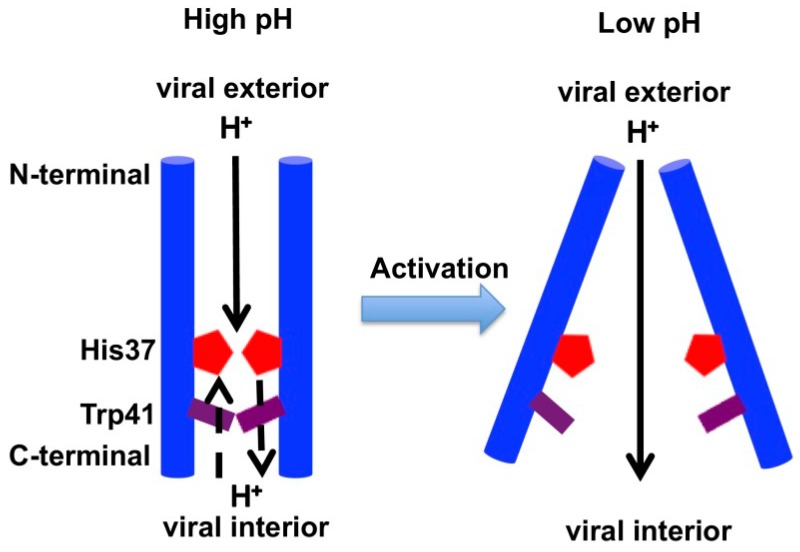
Acid activation mechanism of the AM2 channel. Left: At a high pH (e.g., 7 to 8), the AM2 channel adopts a C_closed_ conformation. The closed Trp41 tetrad dehydrates the His37 tetrad and raises the His37 deprotonation barrier, thereby blocking proton conduction. The low charge state of the His37 tetrad at a high pH reduces the electrostatic repulsion with incoming protons, allowing proton permeation from the viral exterior. Right: At a low pH (below 6), the positive charge on the His37 tetrad increases and the Trp41 gate and C-terminal open and become more hydrated, lowering the His37 deprotonation barrier and increasing proton conductance, thereby leading to channel activation in the C_open_ conformation. Scheme adapted from [70].

At a high pH (e.g., 7 to 8), the AM2 channel is in a C_closed_ conformation (Figure 2, left), where the side chains at the C-terminal end of the channel, including His37 and Trp41 tetrads, are tightly packed, and the pore is lined by alternating layers of side chains and well-ordered water clusters. The closed Trp41 tetrad dehydrates the His37 tetrad and raises the His37 deprotonation barrier, thus blocking proton conduction through the channel. This conformation has been observed by X-ray crystallography [50,51] as well as in both solution and solid-state NMR [52,53,54,55,56]. When the pH decreases to around 6, the His tetrad increases its protonation state to +2, and the channel becomes activated. Electrostatic repulsion with protons is lower due to the low charge state of the His37 tetrad, allowing proton permeation from the viral exterior. This asymmetry partly explains the rectification of proton flow observed experimentally. Protons rapidly diffuse to the His37 tetrad via an ordered water cluster, when the IAV particle is incorporated into the endosome, as it is surrounded by an acidic environment. At a low pH, the positive charge of the His37 tetrad increases, and the Trp41 gate and the C-terminal helices open and become more hydrated. This lowers the His37 deprotonation barrier, increasing proton conductance. Further reduction of pH expands the channel and increases pore water mobility, further increasing proton conductance [57].

The His37 tetrad can adopt four protonation states, one for each His protonation, where the first two protonations already occur at a high pH [58,59]. The transition between C_closed_ to C_open_ state (Figure 2, right) is triggered by protonation of the third His37 at an acidic pH [58,60], with a pKa value that ranges from 4.9 to 6.6 depending on membrane composition [54,58,61,62,63,64]. At this point, protons can be released into the virus interior. This C_open_ form has been characterized by X-ray crystallography [50,51,55,58,65,66,67,68]. Deprotonation of the His37 tetrad triggers a change back to the C_closed_ conformation [55,68]. 

Depending on the role of the His37 tetrad, several proton transport models have been suggested [35]. In the “shutter” mechanism [62], the His37 tetrad works as a gate. At a low pH (around pH 6, see above) the gate opens due to the electrostatic repulsion between the biprotonated, positively charged histidine residues. The excess proton is transferred via the Grotthuss mechanism (i.e., proton transfer through a network of hydrogen-bonded water molecules [69]), without changing the protonation state of His37. In the “shuttle” mechanism (e.g., [55]), the His37 tetrad changes protonation state at a low pH, while the proton is shuttled through the His37–Trp41 region. 

### 2.4. Rate of Proton Conductance in AM2 and BM2

Although the proton conductance rate of AM2 is higher at a low pH [71,72], in the range of 10^1^–10^4^ protons s^−1^ [71,73,74], it is still orders of magnitude lower than expected for a water-filled pore of the size of the M2 channel without ionizable groups [75]. This low conductance may exist to prevent toxicity in the host and has been attributed to a rate-limiting step caused by His37 protonation–deprotonation [36,76]. However, the His37–water proton exchange rate appears to be too fast to account for this (~10^5^ s^−1^) [54], and additional factors may account for the low rate of proton conduction [77,78,79,80]. Recent single-molecule fluorescence experiments using Trp41 as a probe have detected interhelical motion in AM2 on a microsecond timescale, which is consistent with the proton conduction rate, and which increased from a high to low pH with a transition midpoint in the range of those reported for the pKa of the His37 tetrad [81]. This is consistent with a range of studies that show that the AM2 tetramer is more dynamic at a low pH [70,76,77,82,83,84,85,86]. Thus, proton conductance through AM2 may be limited by the transition between C_closed_ and C_open_ conformers. The structure and dynamics of water in the AM2 pore are also important because it is the essential medium for proton permeation. X-ray free-electron laser (XFEL) [68] and 2D infrared (2D-IR) spectroscopy [87] studies of AM2 have shown that, at a low pH, water molecules form a continuous hydrogen-bonded network spanning the channel length. 

In BM2, although conductance at a low pH increases more than for AM2, proton conductance is still much lower than the theoretical conductance at a low pH. This suggests that conformational change of BM2 may also constitute a rate-limiting step [43]. The role of the two His residues, especially His19, is important since an H19C mutation abolishes proton conduction [88], whereas an H27A mutation can reduce proton transport by ∼25% in a liposome proton flux assay [42]. The proton-dissociation equilibrium constants (pKa values) of the His19 tetrad in BM2 TM domain in lipid bilayers were found to be one pH unit lower (i.e., more difficult to protonate) than the pKa values of the AM2-equivalent His37 tetrad [89,90]. This was attributed to the presence of a second titratable histidine, His27 (in the position occupied by Arg45 in AM2), separated by three residues from Trp23 (see Figure 1), forming a putative reverse WxxxH motif. This His27 residue was suggested to increase the proton-dissociation rate of His19 by stabilization of its neutral state. This has been confirmed by a two-electrode voltage clamp (TEVC) electrophysiological assay in *Xenopus* oocytes [43] that compared AM2 (A/Udorn/72) and BM2 (B/Lee/40) (both C-terminally fused to FLAG), where the apparent pK_3_ (3rd protonation state) value of the AM2 channel was about one pH unit higher than that of the BM2 channel, indicating that the latter is less easily protonated and requires a lower pH for activation. Despite this, BM2 was found to have a 10-fold higher conductance than AM2, resulting in an overall 60% higher specific conductance of the BM2 channel than the AM2 channel at a pH of 5.5, which is consistent with liposome flux assays, where the specific conductance of BM2(1–33) is about double that of AM2(18–60) [42]. 

### 2.5. Asymmetric Conductance in AM2 and BM2

The AM2 proton channel is inward rectified (i.e., it conducts more easily in the inward direction), from the N- to C-terminus. This is attributed to Trp41 and Asp44 [37,60]. The Trp41 tetrad forms a gate that physically occludes the C-terminal end of the pore, while Asp44 stabilizes the Trp41 gate via water-mediated hydrogen bonds. When oocytes are subjected to low pH_in_ and high pH_out_ conditions, no outward current in the AM2 proton channel is observed [60]. The W41F mutant of AM2 allows a reverse current when the internal pH was low [59]. The latter ssNMR study showed that the protonation and tautomeric equilibria at His37 were altered, suggesting a role for Trp41 in preventing C-terminal acid activation by His37. 

Interestingly, the BM2 proton channel is slightly outwardly rectified [40]. In BM2, the corresponding residue of AM2-Asp44 is Gly26 (see alignment). Although mutation G26D in BM2 is unable to prevent this outward leakage in a pH_in_ low/pH_out_ high condition, the double mutant G26D/H27R (to mimic AM2 Asp44/Arg45) has achieved similar asymmetric proton flow (I–V curve inward rectified) to the AM2 channel [43]. Thus, asymmetric inward rectified proton flow can be restored by engineering these two residues of AM2 into the corresponding positions of the BM2 channel. Indeed, in the X-ray crystal structure of AM2(22–46), Asp44 participates in ionic interactions with Arg45 and also in water-mediated hydrogen bonding with Trp41 [50]. Conversely, AM2-Asp44 mutants D44N and D44C exhibit an outward current under a low pH_in_ /high pH_out_ condition, similar to BM2 [60].

## 3. CM2 and DM2

### 3.1. CM2

The influenza C virus M2 protein (CM2) was first reported by Hongo et al. [91] and was proposed to function as a voltage-activated chloride ion channel when expressed in *Xenopus* oocytes [92]. CM2 is encoded by RNA segment 6 (M gene) of the influenza C virus [20], which produces a p42 protein precursor, and upon cleavage by a peptidase, CM1 and CM2 are obtained [93,94]. Like AM2, CM2 is a type III membrane protein, with a 23-aa N-terminal extracellular domain, a 23-aa transmembrane domain and a 69-aa C-terminal cytoplasmic domain [95] (Figure 1). CM2 is abundantly expressed in virus-infected cells, and a small amount is incorporated into the virus particles [96]. CM2 forms disulfide-linked dimers and tetramers via evolutionarily conserved Cys1–Cys6–Cys20 [97] and is post-translationally modified by N-glycosylation (Asn11), palmitoylation (Cys65), and phosphorylation [95,96,98]. 

The structure of the TM domain of the CM2 protein (Tyr27–Val46) has been obtained by site-specific infrared dichroism and the molecular modeling [99] of isolate C/Ann Arbor/1/1950 [100]. In that model, side chains of L31, L34, M41 and L44 residues were predicted to be lumenally oriented, and the peptide formed a left-handed coiled-coil tetramer [99,101]. The transmembrane pore of CM2 was occluded by residue M41, and a motif of hydrophilic residues, T30, S33, T40 and Y43, was located at the outer surface of the CM2 channel. 

CM2 protein is capable of modifying pH within the trans-Golgi network (TGN). Indeed, when CM2 was co-expressed with a pH-sensitive hemagglutinin of IAV, CM2 protein was able to protect the co-expressed HA against acid activation in the TGN [102], although its activity was much lower than that of the AM2 protein. Additionally, a chimeric AM2 protein containing the CM2 transmembrane domain could partially restore the infectious virus production of an M2-deficient influenza A virus [103], which suggests that CM2 can alter intracellular pH. Electrophysiological studies of CM2-expressing mouse erythroleukemia cells also identified proton permeability [97], and such permeability probably plays a role in the uncoating process of the influenza C virus. The glycoprotein HEF of the influenza C virus has the ability to cause low pH-dependent hemolysis and fusion [104], and the virion is presumably uncoated in the acidic endosomal compartment. The CM2 protein might, therefore, have a function similar to that of the M2 protein in virus uncoating. Nevertheless, the proton and chloride permeabilities have not been clearly dissected. 

AM2 is 10^6^- to 10^7^-fold more permeable to H^+^ than to alkali metal cations Na^+^/K^+^ [71,105,106]. By contrast, CM2 is permeable to Cl- anions but not to cations, exhibits a milder response to pH [92,102,107] and has only a small proton channel activity that is insensitive to amantadine [92]. Those authors proposed that the CM2 protein is transported to the cell surface where it may lower the ionic strength just beneath the viral budding site by inducing the efflux of Cl- ions. This may be advantageous during virion assembly to promote the interaction of M1 with RNPs, since a slight increase in salt concentration may trigger the dissociation of the M1–RNP complex in ICV [108]. Indeed, deletion of CM2 has caused impaired packaging and uncoating in virus-like particles (VLPs) and recombinant influenza viruses [109]. 

### 3.2. DM2

A new type of influenza virus, known as type D, has recently been identified in cattle and pigs [3]. The proteome of IDV is closer to ICV than to IAV or IBV [2] and, correspondingly, CM2 and DM2 share similarities. DM2 was recently tested in *Xenopus* oocytes using the two-electrode voltage clamp (TEVC) method, and an induced inward current was observed similar to that of CM2, with similar reversal potential [110]. Neither CM2 nor DM2 contain an HxxxW motif (found in AM2 and BM2), but a “YxxxK” motif has been proposed instead [110], where Y and K would have a lumenal orientation (parallel to H and W in the HxxxW motif found in AM2 and BM2) (see alignment). Indeed, the gating voltage of CM2 and DM2 was shown to be affected by modifications at the YxxxK motif [110], which were proposed to be involved in cation–pi pairs between Y and K, and were proposed to be able to regulate ion flow. Overall, the significance of the difference in functional motifs between AM2 and BM2 versus CM2 and DM2 remains to be elucidated. The main features of viroporins in influenza are summarized in Table 1.

## 4. Other Influenza Viroporins

IAV and IBV encode other viroporins, PB1-F2 and NB, respectively. For the sake of completion, these two viroporins are briefly described here. PB1-F2 forms nonselective ion channels in planar lipid bilayers and microsomes [111], and is known to localize to the mitochondria of infected cells [112]. PB1-F2 can interact with two mitochondrial proteins—adenine nucleotide translocator (ANT3) and voltage-dependent anion channel 1 (VDAC1)—present in the inner and outer mitochondrial membranes, respectively, leading to the dissipation of mitochondria membrane potential [113,114]. PB1-F2 from pathogenic IAV can be incorporated into the phagolysosomal compartment to trigger NLRP3 inflammasome activation, inducing the secretion of pyrogenic cytokine IL-1β and thereby leading to severe pathophysiology [115]. In addition, the PB1-F2 of A/Puerto Rico/8/1934(H1N1) (PR8) has been reported to bind the mitochondrial antiviral signaling protein (MAVS), leading to impairment of IFN-β production [114]. However, the precise role of PB1-F2 in modulation of IAV-induced immunopathogenesis remains to be dissected. 

NB protein is encoded by IBV at RNA segment 6 [116]. NB is modified by glycosylation and palmitoylation, with the latter being important for NB trafficking to the cell surface [117]. Purified NB has been found to form ion channels when incorporated into artificial lipid bilayers [118], and further studies on an NB-S20A mutant have resulted in altered proton permeability and channel gating [119]. However, in contrast to the BM2 protein, NB is not required for viral replication in cell culture [120]. IBV NB cannot modify pH within the TGN [107]. 

## 5. Influenza Viroporin Inhibition

The vast majority of viroporin inhibitors have been developed against the AM2 protein. This is not surprising since AM2 was the first viroporin discovered, has a well-established biological role in viral pathogenesis and is a proven drug target [31,38,121]. Amantadine (Amt, Symmetrel) is considered the first viroporin channel inhibitor and the second antiviral agent ever discovered [122]. It binds the pore of the AM2 channel in a hydrophilic pocket [56,123,124]. Together with its close derivative, rimantadine (Rim, Flumadine), they are the only licensed antiviral drugs that target viroporins [125] despite their modest affinity for AM2 (e.g., for Amt, IC_50_ = 16 μM). However, most currently circulating IAV strains are Amt- and Rim-resistant [126,127,128,129], and therefore the use of Amt and Rim has been discontinued in humans [130]. The focus of the current research is the Amt-resistant variants of AM2 [131,132,133,134]. Indeed, more than 95% of circulating influenza A viruses carry the S31N mutation in their M2 sequence, while about 1% have V27A mutations, and less than 0.2% carry rare mutations (L26F, A30T, G34E and L38F) [135]. The reader is referred to previous reviews (e.g., [136,137]) and a more recent one that is also general for viroporin inhibition [138]. Recently, the role of water in drug binding was highlighted by the demonstration that small molecules can enable potent inhibition by targeting key waters in hydrogen-bonded networks that are used to facilitate proton diffusion [51].

Although AM2 and BM2 are functional homologs, wild-type BM2 is insensitive to Amt and Rim [38,40], and no BM2 inhibitors have been identified to date. The lack of amantadine inhibition of BM2 may be explained by differences in lumen hydrophobicity in the two channels. For example, the BM2 structure solved by solution NMR in DHPC [42] shows a coiled-coil tetramer channel apparently in the C_closed_ conformation, with three polar residues (Ser-9, Ser-12, and Ser-16) lining the pore and a bulky phenylalanine ring (Phe-5) protruding into the pore at the N-terminus and possibly blocking the BM2 channel pore. By contrast, the corresponding pore-lining residues in the AM2 channel are much more hydrophobic: Val-27, Ala-30 and Gly-34 [36,53]. Thus, the more hydrophilic channel of BM2 protein may not be able to accommodate a hydrophobic drug like amantadine. However, the poor inhibition by other small molecules is difficult to explain, since N-terminal interhelical separation of BM2, measured using fluorinated Phe-5, cannot prevent the access of small molecules to the lumen of the pore [90]. 

In addition to adamantanes and other small molecules, the AM2 proton channel can be also inhibited by both oxidized and reduced copper ions, Cu(II) and Cu(I). Biphasic inhibition observed in electrophysiological assays has suggested two Cu(II)-binding sites with different affinities [139]. Solid-state NMR studies have shown that the low-affinity site is non-specific and localized at the membrane surface, whereas the high-affinity site would be located inside the channel in between the four imidazole rings from His37 residues and the four indole rings from Trp41 [140]. Influenza A virus replication is affected by Cu(II), presumably through inhibiting the AM2 proton channel by the dissociated Cu(II) [141]. BM2 is also inhibited by Cu(II) in a biphasic manner [43], although it is less sensitive to Cu(II) inhibition. Therefore, Cu(II) complexes may also be toxic to influenza B viruses. 

## 6. M2-Mediated Disruption of Ion Homeostasis 

The channel activity of IAV M2 has been found to be sufficient for the activation of the NLRP3 inflammasome in influenza-infected cells [142]. AM2 also contributes to influenza pathogenesis by downregulating the expression and function of two host ion channels, the amiloride-sensitive epithelial sodium channels (ENaC) [143] and the cystic fibrosis transmembrane conductance regulator (CFTR) chloride channels [144]. These are present in apical membranes of lung epithelium and are important regulators for the absorption and secretion of fluids and electrolytes [145,146]. An impairment in their activity is associated with detrimental consequences, including pulmonary edema and rhinorrhea (see [147]). AM2 has been found to reduce the surface protein levels and channel activity of ENaC, probably via signal transduction mechanisms. M2 can upregulate the steady-state levels of reactive oxygen species, likely by decreasing mitochondrial membrane potential and activation of protein kinase C (PKC), leading to modification of ENaC to enhance its endocytosis and proteasomal degradation. Inhibition of ENaC appears to be mediated by the C-terminus of M2 [143]. 

CFTR is a cyclic AMP-activated chloride channel that mediates the regulation of the thickness and composition of lung epithelial lining fluids (ELFs). M2 reduces the activity of the CFTR channel by alkalinizing the secretory organelle pH, thereby targeting CFTR for destruction via a ubiquitin-dependent pathway. Several studies have demonstrated that the proton channel activity of M2 is required for inhibition of CFTR: (i) addition of M2 ion channel inhibitor amantadine rescued CFTR expression and activity in *Xenopus* oocytes expressing both M2 and CFTR channels, and (ii) co-expression of CFTR with M2 channel-defective mutants (M2-G34V and M2-V27F) did not alter the GlyH-101-sensitive currents [144]. 

## 7. Protein–Protein Interactions (PPIs)

Influenza viroporins are also involved in viral pathogenesis by engaging in critical interactions with viral proteins or disrupting normal host cellular pathways through coordinated interactions with host proteins. The reader is referred to a recent review on this subject [148] that provides an update on the characterization of the main PPIs for most viroporins, as well as the role of viroporins in these PPI interactions. In the next part of this review, we highlight and summarize the nature and outcome of some of these protein–protein interactions involving M2, and how the influenza virus may exploit these host cell factors to its advantage. 

### 7.1. Modulation of Host Autophagy

In IAV, M2 plays an important role in evading host autophagy by interacting with autophagic proteins such as Beclin-1 and LC3 (microtubule-associated protein light chain 3). M2 blocks autophagosome fusion with lysosomes by interacting with Beclin-1 through the N-terminal 60 amino acids of M2, resulting in enhanced apoptotic cell death [149]. IAV is also able to subvert host autophagy by encoding a short linear motif (SLiM) in its M2 protein to mimic a highly conserved LC3-interacting region (LIR) motif, W/FxxI/L/V. This interaction hijacks LC3 to the plasma membrane to prevent autophagosome–lysosome fusion and is essential for IAV budding and transmission [150]. Mutations in M2 that abolish its binding to LC3 have led to reduced filamentous virion budding and stability in vitro [151], suggesting that the M2–LC3 interaction may also aid in viral transmission by enhancing virion stability.

### 7.2. Interplay with Host Defense

Influenza infection induces a number of host antiviral activities, including interferon (IFN) responses and activation of double-stranded RNA (dsRNA)-activated protein kinase (PKR) [152]. During the early stages of infection, host PKR activation is inhibited by IAV to permit viral replication, by (i) masking of viral dsRNA using its nonstructural protein 1 (NS1) to prevent PKR activation [153], and (ii) activation of the PKR inhibitor P58^IPK^ through nucleoprotein (NP)-mediated dissociation of Hsp40 from the Hsp40–P58^IPK^ complex [154]. During the late stages of infection, AM2 stabilizes the Hsp40–P58^IPK^ complex, leading to PKR activation and subsequent cell death and viral release [155]. While BM2 was found to interact with Hsp40 through the C-terminal domain 1 (CTD1) of Hsp40 [155], the binding site in P58^IPK^ is not known.

Tetherin (BST-2, bone stromal cell antigen 2) is an interferon-inducible antiviral host factor that restricts the release of many enveloped viruses by forming a proteinaceous link, tethering budding virions to the cell surface [156]. IAV has evolved several mechanisms to neutralize the activity of tetherin, one of which involves AM2 interaction with tetherin to downregulate its surface expression via the proteasomal pathway, probably mediated by AM2 extracellular and transmembrane domains [157]. 

### 7.3. Targeting by Host Restriction Factors

One identified binder of the cytoplasmic tail of M2 is annexin A6 (AnxA6) [158], a Ca^2+^/lipid-binding scaffold protein that interacts with lipid rafts and regulates cholesterol homeostasis. siRNA-silencing of AnxA6 expression has enhanced viral release, while its overexpression has resulted in reduced viral titer, suggesting that AnxA6 negatively regulates IAV infection [158]. It is speculated that AnxA6 may interact with the M2 cytoplasmic tail during membrane scission, although it remains to be elucidated how this host–pathogen interaction may contribute to the impairment of IAV budding and release. 

Cyclin D3 is a key cell-cycle regulator of the G_0_/G_1_ phase that binds to the M2 cytoplasmic tail [159]. Like AnxA6, siRNA-knockdown of cyclin D3 led to increased virus titer. It has been proposed that cyclin D3 disrupts the interaction between IAV M1 and M2 by competing with M1 for binding to M2, consequently impairing the proper assembly of progeny virions. To counteract cyclin D3 activity, IAV is able to induce the relocalization of cyclin D3 from the nucleus to the cytosol, where it is targeted for proteasomal degradation. 

### 7.4. Modulation of Viral Replication

The cytoplasmic tail region of IAV M2 encodes another host SLiM that interacts with the host caveolin-1 (Cav-1). Cav-1 is a cholesterol-binding raft-residing membrane protein with multiple roles in lipid and membrane trafficking, as well as in signal transduction [160]. Inhibition of Cav-1 correlates with decreased virus titers in IAV-infected cells [161]. A consensus Cav-1-binding motif (CBM), ΦxxxxΦxxΦ (where Φ represents an aromatic amino acid) [162], was found in the amphipathic helix of the M2 cytoplasmic tail (F_47_FKCIYRRF_55_) [161]. The M2/Cav-1 interaction has been found to be involved in modulating IAV replication [161], but it remains to be determined whether this interaction also affects the surface localization of M2 and other viral proteins.

Targeting the function of the host cellular Na,K-ATPase pump offers a promising antiviral treatment (recently reviewed in [163]), since infection by RNA viruses can affect the expression and activity of Na,K-ATPase. For instance, infection with IAV/H1N1 and IAV/H5N1 has led to the downregulation of Na,K-ATPase in alveolar epithelium [164]. Cardiac glycosides are classical inhibitors of Na,K-ATPases and have been reported to exhibit inhibitory effects on the influenza virus [165]. This action of cardiac glycosides on viral replication could be due to activation of signaling cascades, or by altering the concentration of intracellular ions. Influenza protein translation has been reported to be affected by cardiac glycosides such as ouabain, digoxin and lanatoside C, which express antiviral activity against influenza [166,167]. 

The β1 auxiliary subunit of the Na^+^/K^+^ ATPase, ATP1B1, was identified to interact with the cytoplasmic region of both AM2 and BM2, and a stable ATP1B1-knockdown cell line was able to suppress influenza virus A/WSN/33 replication [167]. Using deletion mutations, the authors narrowed down the interacting region to residues 28–48 in BM2, and residues 117–303 in ATP1B1, although the interaction between AM2 and ATP1B1 has not been characterized. 

### 7.5. Modulation of Surface Expression

During the late stages of the IAV replication cycle, the vRNP complexes as well as viral envelope glycoproteins and structural proteins need to be targeted to the virus assembly sites. M2 has to be transported from the rough endoplasmic reticulum (RER) to the apical plasma membrane through the host secretory pathway [168,169]. This transport travelling is thought to be modulated by several cellular factors, including the ubiquitin protein ligase E3 component N-recognin 4 (UBR4), the vesicle transport protein Rab11 and the transport protein particle complex 6A delta (TRAPPC6AΔ). 

UBR4 is a 600-kDa member of the UBR box-containing an N-recognin family with multiple roles, including targeting protein for ubiquitination and proteasomal degradation [170]. In the context of an IAV infection, UBR4 has been proposed to promote surface localization of the viral envelope proteins HA, NA, and M2. The M2 transmembrane and cytoplasmic tail regions have been found to be important for its interaction with host UBR4. Knockout of UBR4 has led to enhanced co-localization of M2 with the autophagosome marker DIRAS3 and reduced M2 cell surface expression [171]. Taken together, the interaction between M2 and host UBR4 may benefit IAV replication by (i) protecting M2 from host degradation, and (ii) promoting surface localization of M2 and other viral glycoproteins to facilitate efficient IAV budding and release. 

The Rab11 pathway is known to be involved in the IAV budding process [172]. Rab11 is a small GTP-binding protein that traffics proteins and vesicles between the trans-Golgi network (TGN), recycling endosome and apical membrane [173,174,175]. Knockout of Rab11 has also led to a reduction of M2 surface levels [176], proposing a role for Rab11 in the apical delivery of M2. 

TRAPPC6A and its truncated form TRAPPC6AΔ also interact with IAV M2 through a highly conserved leucine residue located at the M2 cytoplasmic tail (M2-L96) [177]. TRAPPC6A is a component of the multi-subunit transport protein particle (TRAPP) complex that mediates ER-to-Golgi transport [178]. siRNA knockdown of TRAPPC6AΔ increases M2 surface expression, while TRAPPC6AΔ overexpression has produced the opposite effect, suggesting that TRAPPC6AΔ slows down M2 apical trafficking. Two other subunits of the TRAPP complex, TRAPPC5 and TRAPPC9, were identified as binders to M2 in all of four independent influenza interactome screening campaigns [171,179,180,181]. It would be interesting to determine if these TRAPP complex subunits may cooperate in the modulation of the influenza life cycle. 

Finally, the host factor Golgi-specific brefeldin A-resistant guanine nucleotide exchange factor 1 (GBF1) is proposed to play an important role in the trafficking of viral proteins to the cell surface during the late stages of virus replication [181]. The latter work reported that siRNA-mediated depletion of GBF1 correlates with (i) significant reduction in virus titers, (ii) reduced efficiency of VLP formation and (iii) altered intracellular localization of the viral envelope proteins HA and NA. However, while GBF1 has been reported to associate with M2, it is not completely understood how M2 contributes to the process. 

## 8. Conclusions

In summary, most information available for influenza viroporins is centered around AM2, followed by BM2. For the latter, structural data are increasingly available, but protein–protein interaction data studies comparable to AM2 are lacking. Viroporins in ICV and IDV have attracted much less interest, concomitant with their lower virulence in humans and biomedical relevance. Nevertheless, their mode of action, and those of lesser known viroporins, can help to better understand ionic transport across membranes. 

## Figures and Tables

**Table 1:** Properties of influenza viroporins. PB1-F2 and NB are much less studied compared to M2 proteins, and although studies have been performed, the precise selectivity is not yet clear. The YxxxK motifs in CM2 and DM2 are predicted to be the functional motifs of these channels, although the exact mechanism of action remains to be elucidated.

Influenza Type	Host Range	Viroporin	Coding RNA	Typical Length	Functional Motif	Ion Conductance
A	Humans, aquatic birds, porcine, bovine, canine	AM2 PB1-F2	Segment 7 Segment 2	97 aa 90 aa	HxxxW --	H^+^, K^+^ Ca^2+^, MVC
B	Humans, pigs, harbor seals	BM2 NB	Segment 7 Segment 6	109 aa 100 aa	HxxxW --	H^+^, K^+^ ND
C	Humans, pigs	CM2	Segment 6	115 aa	YxxxK	Cl^−^
D	Cattle, pigs	DM2	Segment 6	152 aa	YxxxK	Cl^−^

Abbreviations: MVC, monovalent cation; ND, not determined.
